# De-Nicotinized Tobacco

**Published:** 1869-02-01

**Authors:** T. Williams

**Affiliations:** Milwaukee


### *De-Nicotinized Tobacco.*

BY T. WILLIAMS, M. D., MILWAUKEE.

It is not generally known that tannic acid de-nicotinizes tobacco. If the bowl of a pipe is filled about one-fourth full of tannin, filled up with tobacco and smoked, the aroma of the tobacco is almost entirely destroyed, and the smoker scarcely feels the effect of the tobacco on his nervous system. In this experiment the tannin powder does not take up all the vaporized nicotine (which is the intoxicating principle of tobacco and tobacco smoke,) as it passes through it. The smoke will at first be entirely free from all taste or smell of tobacco, but in a few moments it will have formed a passage through the tannin, through which it will pass so rapidly that all of its nicotine will not be absorbed.

The experiment is more striking if a bit of sponge is saturated with a saturated solution of tannin, and placed in the bottom of the pipe. The smoke of the first two pipefuls of tobacco will pass out as vaporless and innocent as the smoke of a child's rattan or grape vine cigar, and as devoid of tobacco smell. But if several more pipefuls are smoked, the tannin having taken up all the nicotine it is capable of neutralizing, the smoke will begin to pass out with its natural taste and aroma. A sponge, after being used in this way, acquires a peculiar stale tobacco smoke odor. A common pipe may be used in this experiment, but with it the smoker is very liable to draw some of the tannin solution into his mouth, producing an unpleasant "green persimmon" puckering. The Turkish pipe, which is provided with a reservoir containing water, answers the purpose admirably, The place for water may be filled with a saturated solution of tannin; or what is better,—as it prevents the unpleasant bubbling noise,—a sponge saturated with the solution.

By changing the sponge often enough, a person may smoke as immoderately as he pleases without any injurious effects, and it is particularly recommended to ambitious young gentlemen whom the weed in its natural condition "makes sick." I should also suppose that smoking tobacco steeped in a saturated solution of tannin, and dried, would be equally harmless, but have not tried this latter experiment. I am not sanguine, however, that mankind will avail themselves of the advantages of this discovery. It will be like the Frenchman's antidote to the intoxicating effects of alcoholic potations,—it destroys the very effect for which the poison is used!

The North American Indians were wise, however, and availed themselves of this discovery hundreds of years ago. It is well known what inveterate smokers the Indians are, and still we never see any injurious effects of this habit upon them. This may be due in part to their vigorous constitutions and hardy nomadic life, but it is mainly due, I think, to the form in which they use their tobacco. Until they learn the habit from the whites, they rarely or never use the pure leaf. Their "Killikinnick"—the agreeable aroma of which once inhaled in a wigwam or lumberman's cabin, can never be forgotten,—is composed of equal parts of tobacco and the inside bark of a species of the *cornus coricea.* Sometimes the admixture of tobacco in it is not more than a fourth. This bark is astringent, and abounds in *tannin*, and therefore in a great measure neutralizes the effects of the tobacco. The fancy brands of smoking tobacco labeled "Killickinick," sold by tobacconists in papers, it is needless to say are pure tobacco, and have no real claim to the name. The Indian name for the particular species of swamp dogwood which they use for smoking is "Kinnikinnick," hence the name. As we learned the art of smoking from the American savage, it would be only showing proper respect to our tastes to take the weed as they do. They peel the inside bark of the shrub, dry it, pound it to a powder in their stone mortars, and then mix intimately with the crumbled tobacco.

